# Determining the effect of one decade on fitness of elite Austrian youth soccer players using propensity score matching

**DOI:** 10.3389/fspor.2023.1186199

**Published:** 2023-07-05

**Authors:** Christoph Gonaus, Erich Müller, Thomas Stöggl, Jürgen Birklbauer

**Affiliations:** ^1^ Department of Sport and Exercise Science, University of Salzburg, Salzburg, Austria; ^2^ Department of Science, Analysis and Development, Austrian Football Association, Vienna, Austria; ^3^ Red Bull Athlete Performance Center, Salzburg, Austria

**Keywords:** football, performance development, nearest neighbor matching, statistical control, conditioning tests, talent

## Abstract

Current trends in attacking strategies and increases in external workload have led to a need for fast and well-conditioned athletes in modern soccer. More recently, progressions in speed, coordination, power and endurance were found over a decade in elite Austrian youth players. However, possible confounders such as relative age, maturation, learning effects, and academy philosophy may have influenced these changes. The present study aimed to determine the decade effect on fitness under statistical control of players' exact age, height, body mass, test location as well as total number of pretests and time interval between test and pretest. Players annually completed a battery of anthropometric, general and soccer-specific fitness tests. MANCOVA was calculated to identify the overall impacts of the covariates on fitness. To balance the covariates of initially 2,530 “former” (2002 to 2005) and 2,611 “recent” (2012 to 2015) players, 1:1 nearest neighbor propensity score (PS) matching was used, resulting in 587 U13, 573 U14, 475 U15, 325 U16, 262 U17, and 129 U18 matched pairs. The decade effect on fitness was assessed by independent *t*-tests and Cohen's *d* separately at each age group. Superior performances of recent players were found for linear sprint across all age categories (*d *= 0.154–0.476) as well as for agility (*d *= 0.125–0.340) and change-of-direction speed (*d *= 0.172–0.466) in U15 to U18. Reaction speed increased in U13 (*d *= 0.288) and U15 (*d *= 0.310). Flexibility reduced over the decade in all age categories (*d *= −0.151 to −0.589) and upper-limb power decreased (*d *= −0.278 to −0.347) in U13 and U14. Balancing the covariate distribution via PS matching generally confirmed previous findings, with fitness decade effects reflecting the athletic needs for modern soccer. Since fitness performance changed over time, reference values should be periodically updated. Coaches favor both physical and cognitive fast players nowadays. Thus, training should target all aspects of speed, without disregarding flexibility, upper-limb power and other preventive strategies that keep the players on the pitch.

## 1. Introduction

“Transition is the magic moment in a game” (1) and “pace packs a punch” (2) are two observations in recent technical reports from FIFA World Cup 2014 and UEFA EURO 2020. Current successful teams tend to shift their offensive strategies towards “quick attacks instead of endless elaboration and possession” (3). Referring to English Premier League matches, counterattacks were 3.4 times more effective in creating goal scoring opportunities than combinative play (4). This “vertical mindset” (3) during the transition from defense to offence is characterized by both high ball speed and a low number of passes (5, 6) as well as high sprint speed of the players (7). Associated with this, the goal scoring probability decreases by 7% per additional pass and by 2% per second of attack duration (8). Consequently, modern soccer requires technically and physically skilled players within all playing positions (9).

These trends in attacking strategies are paralleled by greater physical and technical challenges nowadays. Progressions in game speed (i.e., ball speed) and in match structure (i.e., shorter, more intense play periods) (10) as well as increases in external workload (i.e., high-intensity running and sprinting) (11–13) made elite level soccer more physically demanding over the years. In the English Premier League, those increases in workload along with improvements in pitch quality may also have led to more ectomorph players in recent years as they became taller and lighter (14).

Given these trends in body shape and adult match play, it is rational to expect improvements in elite adult and elite youth soccer players' physical fitness over the years. In line with this expectation, sprint performance enhancements have already been found in Norwegian elite adult players (15), and intermittent endurance capacity increased in Dutch U13 to U19 players (16) between the early 90s and late 2000s. On the other hand, power (i.e., countermovement jump) and aerobic capacity (i.e., VO_2max_) of Norwegian elite players (15, 17) as well as height, maturity and functional characteristics (i.e., 10, 20, 40 m sprint, countermovement jump, anaerobic power, VO_2max_, and quadriceps strength) of 13-year-old French academy entrants (18) remained fairly stable during this period. More recently, when comparing elite Austrian youth soccer players' fitness between 2002 to 2005 and 2012 to 2015 seasons, performance increases were found mainly in speed, reaction time, and lower-body power at U13 to U14 age groups as well as in speed, coordination, and endurance at U15 to U18 level (19).

Since these findings in Gonaus et al. (19) were accompanied by changes in anthropometry and birth date distributions and since it is rational to assume that repeated testing and test location biased previous evaluations, it is an important step forward to reanalyze the effect of this 10-year time span (i.e., the effect of the decade) on fitness by best possibly controlling for these confounders. Besides the improvements in fitness, height and body mass increased at younger age groups (i.e., U13 to U14) and a more pronounced relative age effect over the years was present over all age categories, and at U15 level (19). Early born as well as early maturing players may benefit, for example, from temporarily height and weight advantages and superior fitness performance during the talent selection process (20–23), with maturation displaying greater influence on physical performance than relative age (24). In the same vein, even though the criteria for test qualities are fulfilled (25), learning effects resulting from repeated measurements may have influenced the outcome (26). Furthermore, dependent on the academy's philosophy and style of play, different player recruitment criteria and strategies may limit the comparison between players of different academies and talent promotion institutions (27, 28).

To overcome these past limitations, the goal of the current study was to compare the fitness characteristics between former (2002 to 2005) and recent (2012 to 2015) elite Austrian youth soccer players under consideration of the athletes' height, body mass and exact age as well as the total number of pretests, the time interval between pretests and the location of the test. Based on the outlined progression in adult match play and built on our preliminary results, we hypothesized that even under the statistical control of the confounding variables the fitness level in sprint, power and endurance has improved over the investigated decade across age groups (U13 to U18).

## 2. Materials and methods

To test the hypothesis, quasi-longitudinal cohort data from the Austrian soccer talent promotion system were reanalyzed using the propensity score (PS) matching approach to minimize bias in estimation of the decade effect on fitness. The talent promotion system in Austrian soccer was revised in 2001. Since then, U11 to U14 players are selected nationwide and systematically into one of 29 accredited youth development centers (YDC) and the most talented youngsters are subsequently drafted into one of 12 (until 2008: 13) youth soccer academies, covering age groups U15 to U18 (19). In the present analysis, all YDC players at age groups U13 to U14, and all U15 to U18 youth academy players of the seasons 2002 to 2005 and, one decade later, of the seasons 2012 to 2015 were included. These players belong to the highest performance level in their age category in Austria. To monitor the athletic progression during adolescence, all promoted players have to perform a battery of anthropometric, general and soccer-specific fitness tests once, in autumn (U13 to U14), or twice, in summer and winter (U15 to U18), a year. These data were collected within a collaborative project of the Austrian Football Association [Österreichischer Fußball-Bund (ÖFB)], the Department of Sport and Exercise Science of the University of Salzburg and the Elite Sport Centre Austria [Leistungssport Austria; former Institute for Sports Medicine and Science Austria] with the aim to scientifically guide the education and training monitoring in elite Austrian youth soccer players between the ages of 12 to 18 years. When entering the promotion system, all players and their parents or guardians sign a training agreement with the ÖFB, who, for their part, gave their permission to the scientific processing of the data. The study conformed to the Declaration of Helsinki and ethical approval was obtained from the local university ethics committee. Parts of the dataset have been used in two previous studies of Gonaus and Müller (29), and Gonaus et al. (19).

### 2.1. Procedures

To ensure objectivity and standardization, the tests were exclusively conducted by experienced performance diagnosticians and students from either the Department of Sport and Exercise Science of the University of Salzburg, or the Elite Sport Centre Austria on an indoor surface. The time of the day, the sequence of the tests, starting with non-fatiguing exercises, and the measurement systems (30–32) were kept identical throughout the years.

After providing instructions about the test protocol and execution, players' *exact age*, *height* (measured with SECA 217 stadiometer, SECA, Hamburg, Germany), and *body mass* (measured with SECA 813 flat scale, SECA, Hamburg, Germany) were recorded. Following this, the players engaged in a 15 min standardized warm-up, which included running exercises, mobility and activation exercises, as well as sprints. All players consecutively started with one trial of *20 m sprint* and *foot tapping*, subsequently performed two attempts of *sit-and-reach* and *2 kg standing medicine ball throw*, and continued with the second trials of *20 m sprint* and *foot tapping*. Afterwards, they each performed one attempt of *reaction test*, and two trials of 5 × 10 m shuttle sprint, *hurdles agility run*, *countermovement jump* and *drop jump* in a random order. Following a break of 30–45 min, U15 to U18 players conducted the *20 m multi-stage endurance run.* For those tests permitting two attempts, sufficient time to recover was scheduled and only the better score remained for statistical purposes.

### 2.2. Fitness test battery and measurement systems

*5, 10, 20 m sprint:* Linear sprint speed was assessed by *20 m sprint* with 5 and 10 m split times (to the nearest 0.01 s; ICC = 0.561–0.837, Cronbach's Alpha α = 0.762–0.937).

*5 × 10 m shuttle sprint*: Change-of-direction speed was determined by 5 × 10 m shuttle sprint with each 180° turns (0.01 s; ICC = 0.846, α = 0.923).

*Hurdles agility run*: General agility was quantified using the *hurdles agility run* (0.01 s; ICC = 0.793, α = 0.893). A schematic representation of the test setup and the procedure can be found in Gonaus and Müller (29).

Infrared timing gates (Brower Timing Systems, Utah, United States) placed 90 cm above ground were used in all sprint tests and in the agility task. The players started in a step position 0.5 m behind the first timing gate.

*Reaction test*: Lower-limb multi-choice reaction speed was determined by an eye-foot coordination task determining the mean reaction time of 20 stimuli (1 ms; ICC = 0.476, α = 0.698) within four possible directions via the computer-based system of Fitronic (Fitronic Inc., Bratislava, Slovakia).

*Foot tapping*: Maximal speed of lower limbs was assessed using *foot tapping* over 5 s and subsequently calculating bipedal cycles per second (0.1 Hz; ICC = 0.925, α = 0.965).

*20 m multi-stage endurance run*: Aerobic endurance performance was evaluated by the speed corresponding to 4 mmol·l^−1^ (0.1 km·h^−1^; ICC = 0.805, α = 0.916), determined by repetitive 20 m runs for 3 min at 7.92, 9.72, 11.52 and 13.32 km·h^−1^, and blood lactate sampling during 90 s break intervals in between (Lactate Analyzer Biosen 5040, EKF Industrie-Elektronik, Barleben, Germany).

*Countermovement jump*: Lower-body power was evaluated using vertical *countermovement jump* with arm swing (0.1 cm; ICC = 0.749, α = 0.863).

*Drop jump*: Lower-body reactive strength was assessed using two-legged *drop jump* (coefficient 0.01; ICC = 0.744, α = 0.860). The performance from 30 cm (U13 to U14) and 40 cm (U15 to U18) drop height was operationalized by the formula described in Gonaus and Müller (29), taking jump height (0.1 cm) and ground contact time (1 ms) into account.

Both jump tests and the foot tappings were recorded without shoes using a Kistler force plate (Kistler Instrument Corporation, Winterthur, Switzerland).

*2 kg medicine ball throw*: Upper-limb power was determined by an overhead ball throw using a 2 kg medicine ball in size 4 of a football and with a maximum of one-step run-up (0.1 m; ICC = 0.935, α = 0.967).

*Sit-and-reach*: The performance in general flexibility was assessed by the *sit-and-reach* (1 cm; ICC = 0.964, α = 0.990). Positive values indicating that the players reached over their toes while standing on a sit-and-reach box.

A more detailed description of all single tests as well as the reliability analyses of a comparable sample of academy players are provided by Gonaus and Müller (25, 29). According to the principle component analysis in Gonaus and Müller (29), the tests were categorized into “speed” (*5, 10, 20 m sprint*, and *shuttle sprint*), “coordination and endurance” (*agility run*, *reaction test*, *foot tapping*, and *endurance run*), and “power and flexibility” (*countermovement jump*, *drop jump*, *medicine ball throw*, and *sit-and-reach*).

### 2.3. Participants

Analogous to Gonaus et al. (19), the data of 2,530 “former” players tested in the years from 2002 to 2005, and of 2,611 “recent” players tested from 2012 to 2015 were considered. Only the test results from autumn (i.e., September or October; U13 to U14) and winter (i.e., November, December or January; U15 to U18) were regarded. Some players were repeatedly tested during the respective 4-year period, whereas others, due to normal fluctuation within the promotion program, injuries or any other reason for nonparticipation, were investigated less often, leading to a total of 4,058 (2002 to 2005) and 4,448 (2012 to 2015) measurements. Only complete datasets including all anthropometric characteristics and at least 8 out of 9 fitness tests (U13 to U14) or 9 out of 10 tests (U15 to U18) were analyzed; thus, the resulting number of measurements per age group, categorized by period (former vs. recent), were: U13 (*n* = 672 vs. *n* = 964), U14 (713 vs. 906), U15 (570 vs. 869), U16 (427 vs. 740), U17 (357 vs. 482), and U18 (208 vs. 282).

### 2.4. Statistical analyses

Outliers displaying *z*-scores <−4.0 and >4.0 were rejected for each age group and period separately (33).

Some confounding categorical and continuous variables were defined to reduce the bias in the estimation of the decade effect. Categorical variables included were the total number of *pretests* (“0” = no pretest, “1” = 1 pretest, “2” ≥ 2 pretests), the time *interval* between test and pretest (“0” = no pretest, “1” ≤ half a year, “2” ≥ one year), and the *location* of the development center or of the academy (i.e., 9 locations at U13 and U14, 12 locations at U15 to U18). Continuous covariates included were *exact age* (0.01 years), *height* (1 cm), and *body mass* (0.1 kg).

To identify the overall impacts of these confounders on the fitness performance, a multivariate analysis of covariance (MANCOVA) was performed for each age group separately, with *pretests*, *interval* (only U15 to U18), *location*, and *period* as the between-subjects variables, with *exact age*, *height*, and *body mass* as covariates, and with all fitness tests as the dependent variables. Partial eta squared (*η*²_P_) was computed and the significance level was set at *p* < 0.05.

For the matching procedure, a one-dimensional PS was estimated by means of logistic regression (34) using the decade as the outcome variable, and the confounding variables (i.e., covariates) as the predictors to create a matched sample of players from the former period (i.e., 2002 to 2005) and similar sample of players from the recent period (i.e., 2012 to 2015). After calculating the PS for each age group separately, the actual matching on these estimated scores commenced. A 1:1 nearest neighbor matching was applied in order to find the best possible matched groups (35), where each individual of the recent group was matched to that individual of the former group with the respective closest PS. To avoid poor matches, a caliper (i.e., the maximum distance permitted between matched individuals (36) of 0.25 standard deviations of the linear PS (37) was implemented and adjusted for each age group separately (U13 = 0.031; U14 = 0.035; U15 = 0.040; U16 = 0.054; U17 = 0.045; U18 = 0.060). Since each individual can be considered only once for pairing, a random selection order has been selected (38). In a further step, quality checks of the resulting matched samples were assessed by numerical (e.g., standardized differences of the means of the PS), and graphical (e.g., histograms of the PS before and after matching) diagnostics (35, 37, 39). Moreover, chi-squared tests on *pretests*, *interval*, and *location* as well as independent *t*-tests on *PS*, *exact age*, *height*, and *body mass* were conducted to assess the covariate balance before and after matching. Cramer's *V* (*V*) and Cohen's *d* (*d*) were calculated and the significance level was set at *p* < 0.05.

Since small random differences in the confounding variables may still remain even after matching, the subjects should still be treated as independent (40). Thus, and because of lower type-I error, independent samples *t*-tests using a significance level of 0.05 were performed to analyze the effect of the decade on the fitness characteristics. Since the order in which the treated individuals were selected into the matching process has an effect on the quality of the matches (41), the matching procedure, the quality checks and the subsequent inferential statistics (i.e., independent *t*-tests) were repeated 10 times for each age group. Analogous to the pooling phase of the multiple imputation approach described in Enders (42), age-group specific means (i.e., the arithmetic average of the 10 sampling means) and total variances (i.e., summing up the within-imputation variance, the between-imputation variance, and the sampling variance of the mean) were calculated for each four-year period. Cohen's *d* was computed corresponding to the formula for between-subjects designs described in Lakens (43), and was classified as trivial (*d* < 0.2), small (*d* = 0.2), medium (*d* = 0.5), and large (*d* = 0.8), respectively (44).

The PS matching was performed in IBM SPSS statistics version 25.0 (SPSS Inc., Chicago, IL, United States) using the “psmatching 3.04”-program, which is a R plug-in based on the statistical software package R (Version 3.3.3, R Core Team, Auckland, New Zealand). A detailed installation description of “psmatching 3.04” as well as an operation manual for this particular SPSS extension software is described in Thoemmes (34). The subsequent inferential statistics were also executed with IBM SPSS, whereas graphics and descriptive evaluations were generated using Microsoft Office (Version 2016, Microsoft, Seattle, WA, United States).

## 3. Results

The main effects of *pretests*, *interval*, and *location* for all players on the fitness test performance within age group as well as the influence of the covariables *exact age*, *height*, and *body mass* are displayed in Table 1. Main effects were found for *pretests* (*η²_P_* = 0.022–0.086, *p* ≤ 0.038; except for U15-U16), and *location* (*η²_P_ *= 0.021–0.056, *p* < 0.001). Additionally, all covariables (*exact age*, *η²_P_* = 0.019–0.035, *p* ≤ 0.028; *height*, *η²_P_* = 0.041–0.086, *p* ≤ 0.021; *body mass*, *η²_P_* = 0.151–0.293, *p* < 0.001) influenced the fitness performance, except for *exact age* at U17 and U18 level.

**Table 1:** Results of the MANCOVA of all players within age group: effects of pretests, interval, location, and period on fitness performance, controlled for exact age, height, and body mass.

	Fixed factors	Wilks’ lambda	F	df	*p*	*η*²_P_	Covariables	Wilks’ lambda	F	df	*p*	*η*²_P_
U13	Pretests	0.978	3.143	11, 1,557	<0.001	0.022	Exact age	0.965	5.095	11, 1,557	<0.001	0.035
	Location	0.841	3.103	88, 10,219	<0.001	0.021	Height	0.914	13.365	11, 1,557	<0.001	0.086
	Period	0.975	3.665	11, 1,557	<0.001	0.025	Body mass	0.849	25.241	11, 1,557	<0.001	0.151
U14	Pretests	0.914	13.108	11, 1,539	<0.001	0.086	Exact age	0.981	2.734	11, 1,539	0.002	0.019
	Location	0.644	7.957	88, 10,101	<0.001	0.053	Height	0.930	10.463	11, 1,539	<0.001	0.070
	Period	0.854	23.834	11, 1,539	<0.001	0.146	Body mass	0.780	39.394	11, 1,539	<0.001	0.220
U15	Pretests	0.982	1.717	12, 1,125	0.058	0.018	Exact age	0.970	2.912	12, 1,125	0.001	0.030
	Interval	0.986	1.292	12, 1,125	0.217	0.014	Height	0.940	5.937	12, 1,125	<0.001	0.060
	Location	0.663	3.595	132, 9,225	<0.001	0.037	Body mass	0.712	37.964	12, 1,125	<0.001	0.288
	Period	0.968	3.064	12, 1,125	<0.001	0.032						
U16	Pretests	0.983	1.364	12, 973	0.177	0.017	Exact age	0.977	1.930	12, 973	0.028	0.023
	Interval	0.994	0.470	12, 973	0.933	0.006	Height	0.959	3.467	12, 973	<0.001	0.041
	Location	0.739	2.272	132, 7,981	<0.001	0.027	Body mass	0.707	33.671	12, 973	<0.001	0.293
	Period	0.979	1.765	12, 973	0.050	0.021						
U17	Pretests	0.968	1.845	12, 671	0.038	0.032	Exact age	0.981	1.105	12, 671	0.353	0.019
	Interval	0.988	0.670	12, 671	0.782	0.012	Height	0.938	3.707	12, 671	<0.001	0.062
	Location	0.661	2.165	132, 5,509	<0.001	0.037	Body mass	0.765	17.199	12, 671	<0.001	0.235
	Period	0.961	2.268	12, 671	0.008	0.039						
U18	Pretests	0.940	1.965	12, 367	0.026	0.060	Exact age	0.980	0.624	12, 367	0.822	0.020
	Interval	0.964	1.135	12, 367	0.330	0.036	Height	0.938	2.029	12, 367	0.021	0.062
	Location	0.533	1.831	132, 3,021	<0.001	0.056	Body mass	0.826	6.431	12, 367	<0.001	0.174
	Period	0.961	1.246	12, 367	0.250	0.039						

Descriptive and inferential analyses of categorical and continuous covariates within age group in the original dataset as well as in the matched dataset are presented in Tables 2, 3. Differences between former and recent players were found for *pretests* (*V *= 0.071–0.254, *p* ≤ 0.027), *interval* (*V *= 0.085–0.161, *p* ≤ 0.047; except for U18), and *location* (*V *= 0.154–0.355, *p* < 0.001) in the original dataset across all age groups. In addition, pre-matching differences within age group between the two periods were found for *exact age* (U13 to U14, *d* = 0.185–0.303, *p* < 0.001; U15 to U18, *d* = −0.287 to −0.562, *p* < 0.001), *height* (*d* = 0.264–0.320, *p* < 0.001; except for U15 to U18), and *body mass* (U13 to U14, *d* = 0.115–0.183, *p* ≤ 0.022; U16 to U17, *d* = −0.199 to −0.238, *p* ≤ 0.004; except for U15 and U18) as well as for the *propensity score* (*d* = −0.602 to −1.155, *p* < 0.001).

**Table 2:** Descriptive (distribution) and inferential (former vs. recent period) statistics of the categorical covariates before and after propensity score matching.

	Before propensity score matching											After propensity score matching										
	2002 to 2005				2012 to 2015				Chi²	*p*	Cramer's *V*	2002 to 2005				2012 to 2015				Chi²	*p*	Cramer's *V*
U13	*n* = 672				*n* = 964							*n* = 587				*n* = 587						
Pretests	**0**	**1**	** **	** **	**0**	**1**	** **	** **	49.593	<0.001	0.174	**0**	**1**	** **	** **	**0**	**1**	** **	** **	0.004	0.948	0.002
(%)	95	5			84	16						95	5			95	5					
Location (%)	**1**	**2**	**3**	** **	**1**	**2**	**3**	** **	38.978	<0.001	0.154	**1**	**2**	**3**	** **	**1**	**2**	**3**	** **	1.196	0.997	0.032
	12	11	18		9	8	17					11	10	19		12	10	20				
	**4**	**5**	**6**	** **	**4**	**5**	**6**	** **				**4**	**5**	**6**	** **	**4**	**5**	**6**	** **			
	13	9	14		8	13	11					11	10	14		10	10	15				
	**7**	**8**	**9**	** **	**7**	**8**	**9**	** **				**7**	**8**	**9**	** **	**7**	**8**	**9**	** **			
	8	8	7		14	11	10					7	9	8		6	9	8				
U14	*n* = 713				*n* = 906							*n* = 573				*n* = 573						
Pretests	**0**	**1**	** **	** **	**0**	**1**	** **	** **	12.228	<0.001	0.087	**0**	**1**	** **	** **	**0**	**1**	** **	** **	0.019	0.890	0.004
(%)	43	57			35	65						40	60			40	60					
Location (%)	**1**	**2**	**3**	** **	**1**	**2**	**3**	** **	117.686	<0.001	0.270	**1**	**2**	**3**	** **	**1**	**2**	**3**	** **	0.374	1.000	0.018
	11	12	15		12	10	17					13	12	18		13	12	18				
	**4**	**5**	**6**	** **	**4**	**5**	**6**	** **				**4**	**5**	**6**	** **	**4**	**5**	**6**	** **			
	16	11	15		11	16	13					13	13	17		13	13	16				
	**7**	**8**	**9**	** **	**7**	**8**	**9**	** **				**7**	**8**	**9**	** **	**7**	**8**	**9**	** **			
	8	5	7		0	12	10					0	6	8		0	6	8				
U15	*n* = 570				*n* = 869							*n* = 475				*n* = 475						
Pretests	**0**	**1**	**2**	** **	**0**	**1**	**2**	** **	7.219	0.027	0.071	**0**	**1**	**2**	** **	**0**	**1**	**2**	** **	0.201	0.904	0.015
(%)	22	40	38		19	35	46					19	38	42		20	38	42				
Interval	**0**	**1**	**2**	** **	**0**	**1**	**2**	** **	11.863	0.003	0.091	**0**	**1**	**2**	** **	**0**	**1**	**2**	** **	0.203	0.903	0.015
(%)	22	57	21		19	66	15					19	61	20		20	60	19				
Location (%)	**1**	**2**	**3**	**4**	**1**	**2**	**3**	**4**	82.437	<0.001	0.239	**1**	**2**	**3**	**4**	**1**	**2**	**3**	**4**	0.733	1.000	0.028
	2	10	9	5	8	8	9	6				3	10	9	5	3	10	9	4			
	**5**	**6**	**7**	**8**	**5**	**6**	**7**	**8**				**5**	**6**	**7**	**8**	**5**	**6**	**7**	**8**			
	11	11	24	5	9	9	10	9				12	11	18	5	12	11	18	6			
	**9**	**10**	**11**	**12**	**9**	**10**	**11**	**12**				**9**	**10**	**11**	**12**	**9**	**10**	**11**	**12**			
	5	5	10	4	7	8	10	6				6	6	11	4	7	5	10	4			
U16	*n* = 427				*n* = 740							*n* = 325				*n* = 325						
Pretests	**0**	**1**	**2**	** **	**0**	**1**	**2**	** **	73.219	<0.001	0.250	**0**	**1**	**2**	** **	**0**	**1**	**2**	** **	0.022	0.989	0.006
(%)	9	19	71		2	7	91					5	12	83		5	12	83				
Interval	**0**	**1**	**2**	** **	**0**	**1**	**2**	** **	30.084	<0.001	0.161	**0**	**1**	**2**	** **	**0**	**1**	**2**	** **	0.006	0.997	0.003
(%)	9	74	17		2	83	15					5	79	16		5	79	16				
Location (%)	**1**	**2**	**3**	**4**	**1**	**2**	**3**	**4**	77.108	<0.001	0.257	**1**	**2**	**3**	**4**	**1**	**2**	**3**	**4**	0.396	1.000	0.025
	13	11	7	4	8	8	10	7				11	11	8	6	12	11	8	6			
	**5**	**6**	**7**	**8**	**5**	**6**	**7**	**8**				**5**	**6**	**7**	**8**	**5**	**6**	**7**	**8**			
	9	8	21	7	8	9	9	10				10	8	17	7	10	8	16	6			
	**9**	**10**	**11**	**12**	**9**	**10**	**11**	**12**				**9**	**10**	**11**	**12**	**9**	**10**	**11**	**12**			
	3	3	10	4	8	9	10	6				4	4	11	5	4	4	12	5			
U17	*n* = 357				*n* = 482							*n* = 262				*n* = 262						
Pretests	**0**	**1**	**2**	** **	**0**	**1**	**2**	** **	21.433	<0.001	0.160	**0**	**1**	**2**	** **	**0**	**1**	**2**	** **	0.060	0.971	0.011
(%)	6	9	84		3	3	94					4	5	91		5	5	91				
Interval	**0**	**1**	**2**	** **	**0**	**1**	**2**	** **	6.102	0.047	0.085	**0**	**1**	**2**	** **	**0**	**1**	**2**	** **	0.022	0.989	0.006
(%)	6	78	15		3	81	16					4	81	15		5	81	15				
Location (%)	**1**	**2**	**3**	**4**	**1**	**2**	**3**	**4**	39.464	<0.001	0.217	**1**	**2**	**3**	**4**	**1**	**2**	**3**	**4**	0.512	1.000	0.031
	10	11	6	4	9	9	9	5				9	9	8	5	10	10	9	5			
	**5**	**6**	**7**	**8**	**5**	**6**	**7**	**8**				**5**	**6**	**7**	**8**	**5**	**6**	**7**	**8**			
	12	10	19	9	9	10	9	11				11	10	15	10	11	9	14	10			
	**9**	**10**	**11**	**12**	**9**	**10**	**11**	**12**				**9**	**10**	**11**	**12**	**9**	**10**	**11**	**12**			
	3	4	7	6	6	9	10	5				3	5	9	6	4	5	9	6			
U18	*n* = 208				*n* = 282							*n* = 129				*n* = 129						
Pretests	**0**	**1**	**2**	** **	**0**	**1**	**2**	** **	31.533	<0.001	0.254	**0**	**1**	**2**	** **	**0**	**1**	**2**	** **	0.484	0.785	0.043
(%)	2	14	84		2	1	97					3	2	95		2	2	96				
Interval	**0**	**1**	**2**	** **	**0**	**1**	**2**	** **	0.759	0.684	0.039	**0**	**1**	**2**	** **	**0**	**1**	**2**	** **	0.647	0.724	0.050
(%)	2	77	20		2	75	23					3	79	18		2	78	21				
Location (%)	**1**	**2**	**3**	**4**	**1**	**2**	**3**	**4**	61.849	<0.001	0.355	**1**	**2**	**3**	**4**	**1**	**2**	**3**	**4**	1.201	1.000	0.068
	14	9	3	3	12	8	9	2				16	10	3	3	17	8	3	2			
	**5**	**6**	**7**	**8**	**5**	**6**	**7**	**8**				**5**	**6**	**7**	**8**	**5**	**6**	**7**	**8**			
	13	13	16	7	10	7	3	18				14	12	5	10	15	11	7	9			
	**9**	**10**	**11**	**12**	**9**	**10**	**11**	**12**				**9**	**10**	**11**	**12**	**9**	**10**	**11**	**12**			
	3	4	13	2	8	8	9	7				5	6	13	4	5	4	14	5			

Bold values indicate the values of each covariate.

**Table 3:** Descriptive (M ± SD) and inferential (former vs. current period) statistics of the propensity score and the continuous covariates before and after propensity score matching.

	Before propensity score matching					After propensity score matching (pooled estimates)				
	2002 to 2005	2012 to 2015	*t*-statistic	*p*	Cohen's *d*	2002 to 2005	2012 to 2015	*t*-statistic	*p*	Cohen's *d*
U13	*n* = 672	*n* = 964				*n* = 587	*n* = 587			
Propensity score	0.46 ± 0.12	0.38 ± 0.14	−12.286	<0.001	−0.602	0.44 ± 0.12	0.44 ± 0.12	−0.009	0.993	−0.001
Exact age (yrs.)	12.32 ± 0.27	12.42 ± 0.35	6.294	<0.001	0.303	12.33 ± 0.30	12.33 ± 0.34	−0.029	0.977	−0.002
Height (cm)	151.4 ± 7.2	153.7 ± 7.6	6.377	<0.001	0.320	151.8 ± 7.5	151.7 ± 7.6	−0.206	0.837	−0.012
Body mass (kg)	41.0 ± 6.5	42.3 ± 6.8	3.641	<0.001	0.183	41.2 ± 6.8	41.1 ± 6.7	−0.275	0.784	−0.016
U14	*n* = 713	*n* = 906				*n* = 573	*n* = 573			
Propensity score	0.50 ± 0.19	0.39 ± 0.12	−13.093	<0.001	−0.688	0.43 ± 0.11	0.43 ± 0.11	−0.014	0.989	−0.001
Exact age (yrs.)	13.30 ± 0.30	13.35 ± 0.29	3.690	<0.001	0.185	13.33 ± 0.30	13.33 ± 0.33	−0.119	0.905	−0.007
Height (cm)	158.3 ± 8.6	160.5 ± 8.5	5.271	<0.001	0.264	159.2 ± 8.5	159.1 ± 9.0	−0.060	0.952	−0.004
Body mass (kg)	47.0 ± 8.3	47.9 ± 8.2	2.288	0.022	0.115	47.5 ± 8.5	47.3 ± 9.8	−0.263	0.793	−0.016
U15	*n* = 570	*n* = 869				*n* = 475	*n* = 475			
Propensity score	0.47 ± 0.16	0.35 ± 0.16	−13.867	<0.001	−0.747	0.43 ± 0.15	0.43 ± 0.15	−0.007	0.995	0.000
Exact age (yrs.)	14.61 ± 0.37	14.52 ± 0.28	−5.023	<0.001	−0.287	14.59 ± 0.40	14.59 ± 0.29	0.184	0.854	0.012
Height (cm)	169.6 ± 8.2	170.3 ± 7.9	1.457	0.145	0.079	169.7 ± 8.7	169.8 ± 8.5	0.268	0.789	0.017
Body mass (kg)	58.7 ± 9.6	58.1 ± 8.8	−1.275	0.203	−0.069	58.3 ± 10.0	58.5 ± 9.8	0.367	0.714	0.024
U16	*n* = 427	*n* = 740				*n* = 325	*n* = 325			
Propensity score	0.49 ± 0.22	0.29 ± 18	−16.005	<0.001	−1.024	0.41 ± 0.18	0.41 ± 0.18	−0.016	0.987	−0.001
Exact age (yrs.)	15.68 ± 0.29	15.52 ± 0.28	−9.244	<0.001	−0.562	15.63 ± 0.31	15.63 ± 0.27	−0.220	0.826	−0.017
Height (cm)	175.1 ± 6.3	174.8 ± 6.6	−0.748	0.455	−0.045	175.0 ± 6.6	174.9 ± 8.2	−0.225	0.822	−0.018
Body mass (kg)	65.9 ± 7.7	64.0 ± 7.8	−3.910	<0.001	−0.238	65.4 ± 7.9	65.4 ± 10.3	0.048	0.961	0.004
U17	*n* = 357	*n* = 482				*n* = 262	*n* = 262			
Propensity score	0.50 ± 0.18	0.37 ± 0.16	−11.202	<0.001	−0.794	0.44 ± 0.16	0.44 ± 0.16	−0.019	0.985	−0.002
Exact age (yrs.)	16.66 ± 0.29	16.51 ± 0.28	−7.249	<0.001	−0.506	16.60 ± 0.29	16.60 ± 0.26	0.206	0.837	0.018
Height (cm)	177.6 ± 5.8	177.3 ± 6.2	−0.869	0.385	−0.061	177.6 ± 6.3	177.5 ± 7.3	−0.233	0.816	−0.020
Body mass (kg)	69.5 ± 7.2	68.1 ± 7.0	−2.854	0.004	−0.199	69.1 ± 7.5	68.9 ± 7.6	−0.188	0.851	−0.016
U18	*n* = 208	*n* = 282				*n* = 129	*n* = 129			
Propensity score	0.56 ± 0.24	0.32 ± 0.18	−12.133	<0.001	−1.155	0.43 ± 0.19	0.43 ± 0.19	−0.022	0.982	−0.003
Exact age (yrs.)	17.64 ± 0.29	17.50 ± 0.27	−5.313	<0.001	−0.491	17.58 ± 0.30	17.56 ± 0.29	−0.395	0.693	−0.049
Height (cm)	178.5 ± 5.7	178.8 ± 6.1	0.588	0.557	0.054	178.8 ± 6.5	178.7 ± 8.3	−0.127	0.899	−0.016
Body mass (kg)	71.3 ± 6.7	71.0 ± 6.6	−0.417	0.677	−0.038	71.3 ± 7.2	71.4 ± 8.2	0.047	0.963	0.006

The subsequent matching procedure resulted in 587, 573, 475, 325, 262, and 129 players per period in U13, U14, U15, U16, U17, and U18, respectively. After matching, the balance in all categorical covariates (*pretests*, *V* = 0.002–0.043, *p* ≥ 0.785; *interval*, *V* = 0.003–0.050, *p* ≥ 0.724; *location*, *V* = 0.018–0.068, *p* ≥ 0.997) and in all continuous variables (*exact age*, *d* = −0.049–0.018, *p* ≥ 0.693; *height*, *d* = −0.020–0.017, *p* ≥ 0.789; *body mass*, *d* = −0.016–0.024, *p* ≥ 0.714; *PS*, *d* = −0.003–0.000, *p* ≥ 0.982) improved substantially between the two periods throughout all six age groups (Tables 2, 3).

**Figure 1:**
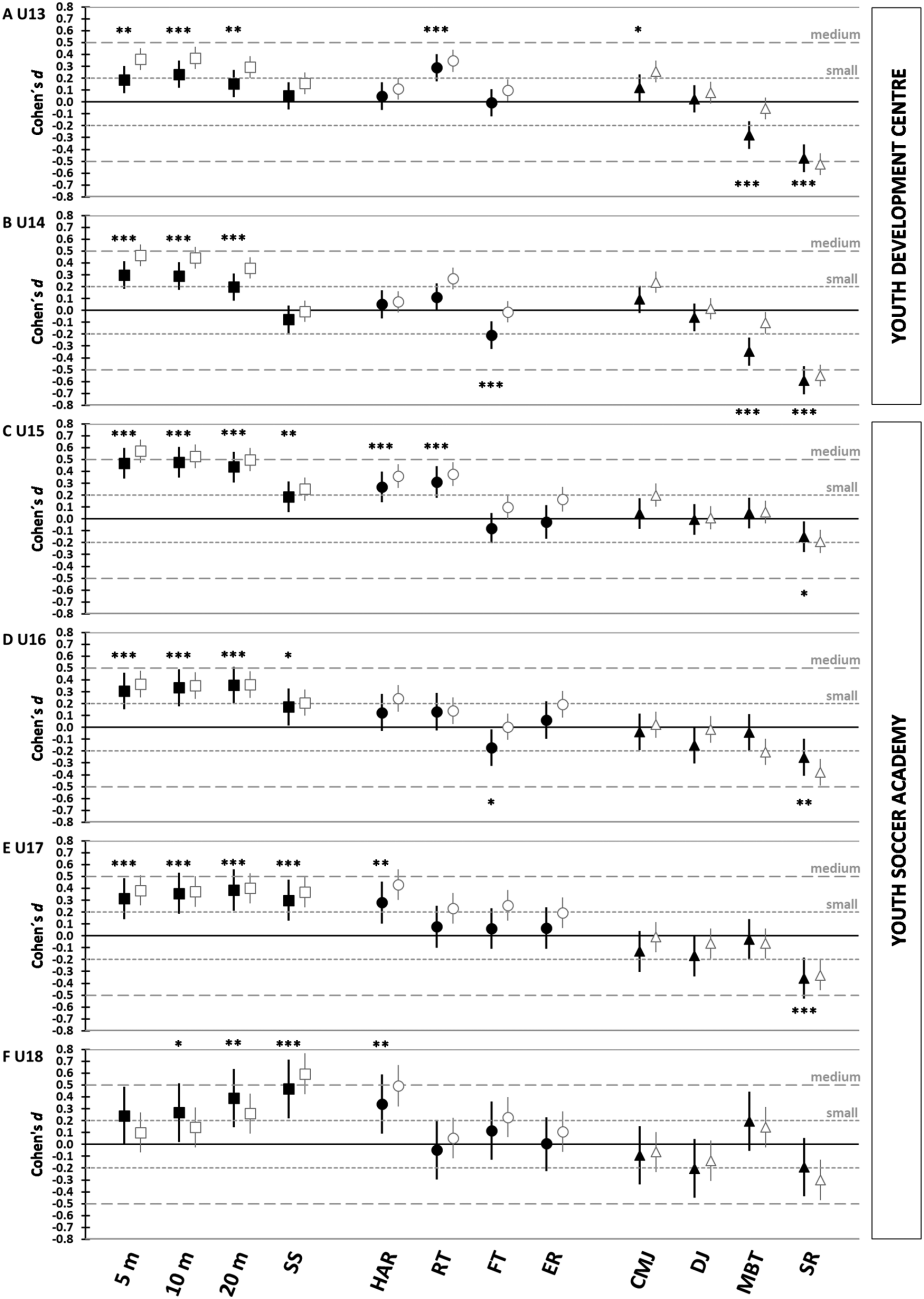
Changes (Cohen's *d*) in “speed” (square), “coordination and endurance” (circle), and “power and flexibility” (triangle) between former (2002 to 2005) and current (2012 to 2015) U13 (**A**), U14 (**B**), U15 (**C**), U16 (**D**), U17 (**E**), and U18 (**F**) soccer players after matching (full symbols) compared to Gonaus et al. (19) (empty symbols). 5/10/20 m = *5/10/20 m sprint*, SS = *5 × 10 m shuttle sprint*, HAR = *hurdles agility run*, RT = *reaction test*, FT = *foot tapping*, ER = *20 m multi-stage endurance run*, CMJ = *countermovement jump*, DJ = *drop jump*, MBT = *2 kg overhead medicine ball throw*, SR = *sit-and-reach*. * *p* < 0.05; ** *p* < 0.01; *** *p* < 0.001. Positive Cohen's *d* values indicate superior performance of current players, whereas negative Cohen's *d* values denote performance decreases.

Figure 1 visualizes performance increases (positive Cohen's *d*), decreases (negative Cohen's *d*) and statistical significances between former (2002 to 2005) and recent (2012 to 2015) Austrian soccer players at YDC (U13 to U14) and academy level (U15 to U18).

### 3.1. Youth development center

Regarding the factor “speed”, after controlling for the covariates, recent YDC players showed better performances in *5 m* (0.01–0.02 s, *d* = 0.187–0.297, *p* ≤ 0.001), *10 m* (0.02–0.03 s, *d* = 0.233–0.290, *p* < 0.001), and *20 m sprint* (0.02–0.03 s, *d* = 0.154–0.196, *p* ≤ 0.008). Only trivial effects of decade were found for *shuttle sprint* (*p* ≥ 0.202) at YDC level (Table 4).

**Table 4:** Descriptive, M ± SD (n), and inferential analyses for the factor “speed”.

	2002 to 2005	2012 to 2015	Mean difference [95% CI]	*t*-statistic	*p*	Cohen's *d* [95% CI]
5 m sprint (s)						
U13	1.17 ± 0.07 (584)	1.16 ± 0.06 (584)	0.01 [0.00; 0.02]	3.197	0.001	0.187 [0.072; 0.302]
U14	1.14 ± 0.07 (573)	1.12 ± 0.06 (573)	0.02 [0.01; 0.03]	5.021	<0.001	0.297 [0.180; 0.413]
U15	1.10 ± 0.07 (475)	1.06 ± 0.06 (475)	0.03 [0.02; 0.04]	7.191	<0.001	0.467 [0.338; 0.596]
U16	1.06 ± 0.07 (325)	1.04 ± 0.06 (325)	0.02 [0.01; 0.03]	3.904	<0.001	0.306 [0.152; 0.461]
U17	1.05 ± 0.07 (262)	1.03 ± 0.06 (262)	0.02 [0.01; 0.03]	3.578	<0.001	0.313 [0.140; 0.485]
U18	1.04 ± 0.06 (129)	1.03 ± 0.05 (129)	0.01 [−0.00; 0.03]	1.934	0.054	0.241 [−0.004; 0.486]
10 m sprint (s)						
U13	2.01 ± 0.10 (584)	1.99 ± 0.09 (584)	0.02 [0.01; 0.03]	3.976	<0.001	0.233 [0.118; 0.348]
U14	1.96 ± 0.09 (573)	1.93 ± 0.09 (573)	0.03 [0.02; 0.04]	4.902	<0.001	0.290 [0.173; 0.406]
U15	1.87 ± 0.10 (475)	1.83 ± 0.08 (475)	0.04 [0.03; 0.05]	7.328	<0.001	0.476 [0.346; 0.604]
U16	1.81 ± 0.09 (325)	1.79 ± 0.08 (325)	0.03 [0.02; 0.04]	4.254	<0.001	0.334 [0.179; 0.489]
U17	1.79 ± 0.09 (262)	1.76 ± 0.07 (262)	0.03 [0.01; 0.04]	4.101	<0.001	0.358 [0.185; 0.531]
U18	1.77 ± 0.07 (129)	1.76 ± 0.06 (129)	0.02 [0.00; 0.04]	2.143	0.033	0.267 [0.021; 0.512]
20 m sprint (s)						
U13	3.51 ± 0.16 (584)	3.49 ± 0.15 (584)	0.02 [0.01; 0.04]	2.638	0.008	0.154 [0.039; 0.269]
U14	3.41 ± 0.16 (573)	3.38 ± 0.16 (573)	0.03 [0.01; 0.05]	3.327	<0.001	0.196 [0.080; 0.312]
U15	3.24 ± 0.16 (475)	3.17 ± 0.15 (475)	0.07 [0.05; 0.09]	6.736	<0.001	0.437 [0.308; 0.566]
U16	3.12 ± 0.13 (325)	3.08 ± 0.15 (325)	0.05 [0.03; 0.07]	4.534	<0.001	0.356 [0.201; 0.511]
U17	3.07 ± 0.12 (262)	3.03 ± 0.11 (262)	0.04 [0.02; 0.06]	4.409	<0.001	0.385 [0.212; 0.558]
U18	3.05 ± 0.11 (129)	3.01 ± 0.10 (129)	0.04 [0.02; 0.07]	3.130	0.002	0.390 [0.143; 0.636]
5 × 10 m shuttle sprint (s)						
U13	12.75 ± 0.57 (585)	12.72 ± 0.57 (585)	0.03 [−0.03; 0.09]	0.898	0.369	0.053 [−0.062; 0.167]
U14	12.34 ± 0.53 (570)	12.38 ± 0.55 (570)	−0.04 [−0.10; 0.02]	−1.275	0.202	−0.076 [−0.192; 0.041]
U15	11.77 ± 0.52 (470)	11.68 ± 0.48 (470)	0.09 [0.03; 0.16]	2.850	0.004	0.186 [0.058; 0.314]
U16	11.42 ± 0.47 (324)	11.34 ± 0.45 (324)	0.08 [0.01; 0.15]	2.184	0.029	0.172 [0.017; 0.326]
U17	11.27 ± 0.41 (260)	11.15 ± 0.41 (260)	0.12 [0.05; 0.19]	3.405	<0.001	0.299 [0.126; 0.472]
U18	11.22 ± 0.41 (129)	11.02 ± 0.45 (129)	0.20 [0.09; 0.31]	3.740	<0.001	0.466 [0.218; 0.713]

With respect to “coordination”, trivial to small enhancements of recent YDC players were reported for *reaction test* (11–29 ms, *d* = 0.111–0.288, *p* ≤ 0.062). Trivial changes over the decade were computed for *hurdles agility run* (*p* ≥ 0.391) at U13 to U14 and *foot tapping* (*p* = 0.909) at U13. The latter even displayed a performance decrease over the years at U14 (−0.2 Hz, *d* = −0.208, *p* < 0.001) (Table 5).

**Table 5:** Descriptive, M ± SD (n), and inferential analyses for the factor “coordination and endurance”.

	2002 to 2005	2012 to 2015	Mean difference [95% CI]	*t*-statistic	*p*	Cohen's *d* [95% CI]
Hurdles agility run (s)						
U13	12.95 ± 0.93 (582)	12.90 ± 0.98 (582)	0.05 [−0.06; 0.15]	0.834	0.404	0.049 [−0.066; 0.164]
U14	12.57 ± 0.82 (565)	12.52 ± 0.99 (565)	0.05 [−0.06; 0.15]	0.857	0.391	0.051 [−0.066; 0.168]
U15	12.02 ± 0.75 (468)	11.82 ± 0.75 (468)	0.20 [0.11; 0.29]	4.112	<0.001	0.269 [0.140; 0.398]
U16	11.53 ± 0.66 (316)	11.45 ± 0.75 (316)	0.09 [−0.02; 0.20]	1.566	0.118	0.125 [−0.032; 0.281]
U17	11.37 ± 0.61 (251)	11.18 ± 0.71 (251)	0.19 [0.07; 0.30]	3.133	0.002	0.280 [0.104; 0.455]
U18	11.30 ± 0.70 (125)	11.06 ± 0.69 (125)	0.23 [0.06; 0.41]	2.682	0.008	0.340 [0.089; 0.589]
Reaction test (ms)						
U13	760 ± 105 (585)	731 ± 93 (585)	29 [17; 40]	4.927	<0.001	0.288 [0.173; 0.403]
U14	686 ± 92 (567)	675 ± 104 (567)	11 [−1; 23]	1.867	0.062	0.111 [−0.006; 0.227]
U15	628 ± 92 (440)	603 ± 70 (440)	25 [15; 36]	4.590	<0.001	0.310 [0.177; 0.442]
U16	578 ± 79 (306)	569 ± 60 (306)	9 [−1; 20]	1.643	0.101	0.133 [−0.026; 0.291]
U17	563 ± 75 (248)	558 ± 57 (248)	5 [−6; 17]	0.856	0.392	0.077 [−0.099; 0.253]
U18	551 ± 62 (124)	554 ± 61 (124)	−3 [−18; 12]	−0.354	0.724	−0.045 [−0.294; 0.204]
Foot tapping (Hz)						
U13	11.1 ± 1.1 (585)	11.0 ± 1.2 (585)	−0.0 [−0.1; 0.1]	−0.114	0.909	−0.007 [−0.121; 0.108]
U14	11.7 ± 1.1 (572)	11.5 ± 1.2 (572)	−0.2 [−0.4; −0.1]	−3.513	<0.001	−0.208 [−0.324; −0.091]
U15	12.7 ± 1.3 (475)	12.6 ± 1.2 (475)	−0.1 [−0.3; 0.1]	−1.216	0.224	−0.079 [−0.206; 0.048]
U16	13.4 ± 1.2 (323)	13.2 ± 1.2 (323)	−0.2 [−0.4; −0.0]	−2.165	0.031	−0.170 [−0.325; −0.016]
U17	13.6 ± 1.3 (262)	13.7 ± 1.2 (262)	0.1 [−0.1; 0.3]	0.710	0.478	0.062 [−0.109; 0.233]
U18	13.7 ± 1.2 (129)	13.9 ± 1.4 (129)	0.2 [−0.2; 0.5]	0.925	0.356	0.115 [−0.129; 0.360]
20 m multi-stage endurance run (km h^−1^)						
U15	11.92 ± 0.85 (384)	11.89 ± 0.70 (384)	−0.02 [−0.13; 0.09]	−0.364	0.716	−0.026 [−0.168; 0.115]
U16	12.01 ± 0.84 (312)	12.06 ± 0.70 (312)	0.05 [−0.08; 0.17]	0.767	0.443	0.061 [−0.096; 0.218]
U17	12.15 ± 0.88 (249)	12.20 ± 0.71 (249)	0.05 [−0.09; 0.20]	0.729	0.466	0.065 [−0.110; 0.241]
U18	12.22 ± 0.72 (119)	12.23 ± 0.69 (119)	0.00 [−0.17; 0.18]	0.042	0.966	0.005 [−0.224; 0.229]

Concerning “power and flexibility”, former YDC players outperformed the recent players in the *medicine ball throw* (−0.3 m to −0.5 m, *p* < 0.001) and *sit-and-reach* (−2.8 to −3.2 cm, *p* < 0.001), with small effects in upper-limb power (*d* = −0.278 to −0.347) and medium effects in flexibility (*d* = −0.474 to −0.589). Only trivial effects of decade were measured in *countermovement jump* (*p* ≥ 0.044) and *drop jump* (*p* ≥ 0.314) in both age groups (Table 6).

**Table 6:** Descriptive, M ± SD (n), and inferential analyses for the factor “power and flexibility”.

	2002 to 2005	2012 to 2015	Mean difference [95% CI]	*t*-statistic	*p*	Cohen's *d* [95% CI]
Countermovement jump (cm)						
U13	26.7 ± 5.2 (587)	27.3 ± 4.9 (587)	0.6 [0.0; 1,2]	2.019	0.044	0.118 [0.003; 0.232]
U14	29.1 ± 5.8 (572)	29.6 ± 5.1 (572)	0.5 [−0.1; 1.1]	1.569	0.117	0.093 [−0.023; 0.209]
U15	33.9 ± 5.6 (475)	34.2 ± 5.5 (475)	0.2 [−0.5; 1.0]	0.689	0.491	0.045 [−0.083; 0.172]
U16	37.0 ± 5.4 (325)	36.8 ± 6.5 (325)	−0.2 [−1.1; 0.6]	−0.479	0.632	−0.038 [−0.191; 0.116]
U17	38.7 ± 5.7 (262)	38.0 ± 5.1 (262)	−0.7 [−1.6; 0.2]	−1.499	0.134	−0.131 [−0.302; 0.040]
U18	39.5 ± 5.2 (129)	39.0 ± 5.3 (129)	−0.5 [−1.8; 0.8]	−0.749	0.455	−0.093 [−0.338; 0.151]
Drop jump (coeff.)						
U13	5.05 ± 1.80 (583)	5.10 ± 2.06 (583)	0.05 [−0.17; 0.27]	0.422	0.673	0.025 [−0.090; 0.139]
U14	5.70 ± 1.89 (573)	5.58 ± 2.16 (573)	−0.12 [−0.35; 0.11]	−1.008	0.314	−0.060 [−0.175; 0.056]
U15	6.48 ± 2.27 (468)	6.47 ± 2.00 (468)	−0.01 [−0.27; 0.25]	−0.092	0.927	−0.006 [−0.134; 0.122]
U16	7.49 ± 2.21 (319)	7.14 ± 2.49 (319)	−0.35 [−0.73; 0.02]	−1.900	0.058	−0.150 [−0.306; 0.005]
U17	8.33 ± 2.21 (259)	7.94 ± 2.44 (259)	−0.39 [−0.79; 0.01]	−1.901	0.058	−0.167 [−0.340; 0.006]
U18	8.55 ± 2.85 (126)	8.02 ± 2.36 (126)	−0.53 [−1.16; 0.10]	−1.611	0.109	−0.203 [−0.451; 0.045]
2 kg overhead medicine ball throw (m)						
U13	6.0 ± 1.3 (580)	5.7 ± 1.0 (580)	−0.3 [−0.4; −0.2]	−4.739	<0.001	−0.278 [−0.394; −0.163]
U14	7.1 ± 1.6 (567)	6.6 ± 1.3 (567)	−0.5 [−0.7; −0.3]	−5.850	<0.001	−0.347 [−0.465; −0.230]
U15	8.6 ± 1.5 (470)	8.7 ± 1.7 (470)	0.1 [−0.1; 0.3]	0.757	0.449	0.049 [−0.078; 0.177]
U16	9.9 ± 1.4 (323)	9.9 ± 1.8 (323)	−0.1 [−0.3; 0.2]	−0.524	0.600	−0.041 [−0.195; 0.113]
U17	10.7 ± 1.5 (261)	10.6 ± 1.5 (261)	−0.0 [−0.3; 0.2]	−0.350	0.727	−0.031 [−0.202; 0.141]
U18	11.0 ± 1.4 (126)	11.3 ± 1.7 (126)	0.3 [−0.1; 0.7]	1.544	0.124	0.194 [−0.053; 0.441]
Sit-and-reach (cm)						
U13	6.4 ± 5.2 (583)	3.7 ± 6.5 (583)	−2.8 [−3.5; −2.1]	−8.091	<0.001	−0.474 [−0.590; −0.357]
U14	8.1 ± 5.4 (573)	4.9 ± 5.5 (573)	−3.2 [−3.8; −2.6]	−9.973	<0.001	−0.589 [−0.707; −0.471]
U15	10.0 ± 6.4 (474)	9.0 ± 7.3 (474)	−1.0 [−1.9; −0.2]	−2.328	0.020	−0.151 [−0.279; −0.024]
U16	12.8 ± 6.4 (322)	11.0 ± 8.0 (322)	−1.8 [−2.9; −0.7]	−3.204	0.001	−0.253 [−0.408; −0.097]
U17	13.5 ± 6.3 (260)	11.2 ± 6.9 (260)	−2.4 [−3.5; −1.2]	−4.065	<0.001	−0.357 [−0.530; −0.183]
U18	13.4 ± 6.3 (128)	12.1 ± 7.1 (128)	−1.3 [−2.9; 0.3]	−1.542	0.124	−0.193 [−0.439; 0.053]

### 3.2. Youth soccer academy

With reference to “speed”, recent academy players outperformed the former players within all age groups at *5 m* (0.01–0.03 s, *d* = 0.241–0.467, *p* ≤ 0.054), *10 m* (0.02–0.04 s, *d* = 0.267–0.476, *p* ≤ 0.033), *20 m* (0.04–0.07 s, *d* = 0.356–0.437, *p* ≤ 0.002), and *shuttle sprint* (0.08–0.20 s, *d* = 0.172–0.466, *p* ≤ 0.029). The largest effect sizes were detected at U15 level for the *linear sprint* (*5 m*, *d* = 0.467; *10 m*, *d* = 0.476; *20 m*, *d* = 0.437), and at U18 for the *shuttle sprint* (*d* = 0.466) (Table 4).

Regarding “coordination and endurance”, *hurdles agility run* improved over the decade across all age groups (0.19–0.23 s, *d* = 0.269–0.340, *p* ≤ 0.008), except for U16 (*p* ≥ 0.118), whereas *reaction test* performance enhanced solely at U15 (25 ms, *d* = 0.310, *p* < 0.001). Only trivial effects over time were found for *foot tapping* (*d* = −0.170–0.115, *p* = 0.031–0.478) and *endurance run* (*p* ≥ 0.443) at academy level (Table 5).

With respect to “power and flexibility”, former players were more flexible across academy years (−1.0 to −2.4 cm, *p* ≤ 0.020; except for U18), with *d* = −0.151 to −0.357. In addition, trivial to small performance decreases were shown in *drop jump* at U16 to U18 (−0.35 to −0.53, *d* = −0.150 to −0.203, *p* ≤ 0.109). Only trivial effects over the decade were detected in *countermovement jump* (*p* ≥ 0.134) and *medicine ball throw* (*p* ≥ 0.124) across all academy age groups (Table 6).

## 4. Discussion

We evaluated whether the fitness level of elite Austrian youth soccer players has changed over one decade under statistical control for players' height, body mass and exact age as well as the total number of pretests, the time interval between pretests and the location of the test. Superior performances of recent players were found for linear sprint speed across all age categories as well as for general agility and change-of-direction speed at academy level. In addition, reaction speed increased over the decade most notably at U13 and U15 level. However, flexibility decreased over time in almost all age categories and upper-limb power decreased at YDC level.

The improvements of sprint speed in elite Austrian youth soccer players over the years fit well into current prospects that soccer “is likely to be played at higher speeds in the future” (45). Evolutions in game speed and the shift to shorter, more intense play periods (10) as well as enhancements of maximum running speed and higher proportions of explosive sprints (11) underline that a certain level of sprint speed is indispensable within the modern game. Besides linear sprint, the observed enhancements at academy level in general agility and in the ability to perform rapid changes in direction may be ascribed to progresses concerning training specificity (46) as contemporary training approaches integrate physical work into tactical and technical work by the use of small sided games (47) and soccer-specific change-of-direction drills (48, 49). Nevertheless, supplementing small sided games and soccer-specific drills with isolated strength, power and speed training may be most beneficial during the academy years (50, 51) to enhance both the ability to perform single maximal efforts (i.e., acceleration, deceleration, change-of-direction) and the ability of repeating such maximal efforts (52). The prognostic relevance of both linear and change-of-direction speed as well as repeated sprint ability and endurance in terms of talent identification (53) further underlines the need for both fast and well-conditioned players within the modern game.

Furthermore, recent players showed superior multi-choice reaction time performance over the years, especially at U13 and U15 level. These two age groups mark decisive transitions within the Austrian talent promotion system, where players are selected into either YDC (U13) or youth soccer academies (U15). Growing evidence and awareness of the impact of cognitive performance on the success in ball sports (e.g., 54) make it very plausible that coaches favor players with improved cognitive functions (e.g., faster decision making skills) and better game reading skills (e.g., responding more rapidly to a relevant sign) nowadays (10, 55, 56). Besides these positive performance trends over the investigated period, recent players demonstrate inferior general flexibility compared to former ones, ranging from small to medium Cohen's *d* throughout all age categories. Considering that a reduced hip flexion range of motion increases the likelihood for hamstring injuries (57) and that hamstring flexibility is a key factor for performing soccer-specific skills (58), dynamic warm-up programs including strength, balance and mobility exercises should be added before games or during training sessions to counter this negative trend (59).

Within the context of selection policies, relative age and biological maturation are often attributed to affect fitness performance at young ages (20, 21, 60) and thus, to influence selection decisions in favor of early born or, even more, early-matured players in elite youth soccer (61).

However, it is important to note that the impact of biological maturation is greater on fitness than on motor coordination skills (62) and that the effect of relative age and maturation should be recognized as independent constructs (63). Accordingly, the present MANCOVA showed a significant influence of the covariables *exact age, height* and *body mass* on the fitness performance. In addition, the number of *pretests*, e.g., representing potential learning effects (26) as well as the *location* of the YDC or academy, e.g., that coaches use different player recruitment criteria and strategies (28), significantly influenced the fitness outcome. Thus, the results of the MANCOVA served as the rational for controlling these variables via statistical matching.

The results of the subsequent PS matching showed that the matching led to an improved balance in all confounding variables (*exact age, height, body mass, pretests, interval, location*) between the two periods throughout all six age groups. Any statistical pre-matching differences of these confounders disappeared after the matching procedure. Appling the PS matching approach is rather unique in the talent development research, even though this established multivariate matched sampling method has received increased attention in medical research (64) and social sciences (65). The statistical procedure is typically applied in observational studies, when random assignment to condition is not feasible (66). It uses the PS as a single balancing variable to construct probabilistically equivalent groups on the relevant covariates (39), and thus to minimize the bias in the estimation of the treatment effect (35).

Compared to the original, non-matched dataset in Gonaus et al. (19), balancing this covariate distribution via PS matching resulted in rather similar outcomes. Even after controlling for *exact age, height, body mass, pretests, interval,* and *location*, recent Austrian youth soccer players were faster but less flexible than former players in all age groups. However, the size of the decade effects decreased especially at younger age groups when comparing the non-matched vs. the PS matched dataset: at U13 to U14 in *5 m* (0.360–0.463 vs. 0.187–0.297), *10 m* (0.371–0.443 vs. 0.233–0.290), and *20 m sprint* (0.294–0.358 vs. 0.154–0.196) as well as at U13 to U15 in *countermovement jump* (0.199–0.255 vs. 0.045–0.118). It is reasonable that the greater influence of the covariates on speed and lower-body power in younger age groups is primarily caused by age and maturity related factors, underpinning the importance to take these in talent identification and long-term analyses of fitness parameters into account, particularly at YDC level. Similar effects of the decade were found for reaction time at younger age groups (U13 and U15) and for general agility as well as for change-of-direction speed at academy level.

The present analysis is based upon our data from 2019 (19) but aims to best possibly determine the effect of the decade on fitness test performance alone by taking into account for some important limitations mentioned in our previous paper. It is one main strength of our study that it draws on a comprehensive fitness test battery and on a large, longitudinal and nationwide sample. A second unique feature is that the PS matching approach was applied to reduce the bias in the estimation of the decade effect on fitness when controlling for exact age, anthropometric variables, repeated testing and academy location. Unlike traditional parametric models such as the analysis of covariance, PS matching does not rely on strict assumptions about the data (67) and is capable to simultaneously control for many covariates (68). It has further advantages over alternative approaches to achieve balance in the covariate distribution particularly when conditions or groups do not fully overlap, and there are nonlinear relationships between covariates and the outcome (39).

Nevertheless, supplementary information on training content and the amount of training hours might have been beneficial to draw more precise conclusions about whether performance improvements over time can be attributed to training induced evolutions or simply to selection modifications. In addition, even though height and body mass were included as confounders, more specific conclusions about the impact of maturity on the current results may have been achieved with knowledge of the level of maturation. For the future, adding another decade (i.e., seasons 2022 to 2025) would be of great interest to further examine the fitness evolution in elite Austrian youth soccer players. Also, assuming that a sufficient amount of data are available, the athletic development from U13 to U18 should be presented in a true longitudinal design and could be further compared over the decades. The prerequisite for this approach, however, is to maintain consistency in the tests and the test procedures throughout the years. Another limitation of the current procedure might be that all tests are performed on the same day. Nevertheless, this approach is common for field-based test batteries to balance testing economy (i.e., time efficiency) and logistical factors (i.e., player availability), provided that a sufficient amount of recovery between the tests and a standardized test protocol is ensured (69).

## 5. Conclusion

Along with the evolution of physical performance in professional soccer, elite Austrian youth soccer players have become faster over a 10-year period under statistical control for exact age, anthropometric variables, repeated testing and academy location. These progressions in speed were not only restricted to advances in linear sprint speed and change-of-direction ability but also to improvements in reaction time. Soccer training should therefore target all aspects of speed, both the physical as well as the cognitive component. Preferable training contents should include small sided games and soccer-specific drills but also consist of isolated strength, power and speed training. Cognitive components should be improved by appropriate training interventions and talent diagnostics should be upgraded by adding cognitive tests. To prepare the youth soccer players optimally for the transition to the first-team and to keep up with the ongoing progressions of the elite level game demands, reference values of fitness tests in youth soccer should be updated on a regular basis. Besides those activities to enhance players' performance, flexibility training along with other preventive strategies to avoid injuries should not be neglected in order to maintain and/or increase players’ availability on the pitch.

## Data Availability

The raw data supporting the conclusions of this article will be made available by the authors, without undue reservation.

## References

[1] Fifa. “Technical Report and Statistics: 2014 FIFA World Cup Brazil”. (Zurich) (2014).

[2] Uefa. “Technical Report: UEFA Euro 2020”. (Nyon) (2021).

[3] Fifa. “Technical Report: FIFA U-20 World Cup Poland 2019”. (Zurich) (2019).

[4] Gonzalez-Rodenas J Aranda-Malaves R Tudela-Desantes A Nieto F Uso F Aranda R. Playing tactics, contextual variables and offensive effectiveness in English premier league soccer matches. A multilevel analysis. PLoS One. (2020) 15:e0226978. 10.1371/journal.pone.022697 32069336 PMC7028361

[5] Tenga A Sigmundstad E. Characteristics of goal-scoring possessions in open play: comparing the top, in-between and bottom teams from professional soccer league. Int J Perf Analy in Sport. (2011) 11:545–52. 10.1080/24748668.2011.11868572

[6] Wright C Atkins S Polman R Jones B Sargeson L. Factors associated with Goals and Goal Scoring Opportunities in Professional Soccer. Int J Perfor Analy in Sport. (2011) 11: 438–49.

[7] Hewitt A Greenham G Norton K. Game style in soccer: what is it and can we quantify it? Int J Perfor Analy in Sport. (2016) 16:355–72. 10.1080/24748668.2016.11868892

[8] Sarmento H Anguera MT Pereira A Araujo D. Talent identification and development in male football: a systematic review. Sports Med. (2018) 48:907–31. 10.1007/s40279-017-0851-7 29299878

[9] Bush M Barnes C Archer DT Hogg B Bradley PS. Evolution of match performance parameters for various playing positions in the English premier league. Hum Mov Sci. (2015) 39:1–11. 10.1016/j.humov.2014.10.003 25461429

[10] Wallace JL Norton KI. Evolution of world cup soccer final games 1966-2010: game structure, speed and play patterns. J Sci Med Sport. (2014) 17:223–8. 10.1016/j.jsams.2013.03.016 23643671

[11] Barnes C Archer DT Hogg B Bush M Bradley PS. The evolution of physical and technical performance parameters in the English premier league. Int J Sports Med. (2014) 35:1095–100. 10.1055/s-0034-1375695 25009969

[12] Zhou C Gomez MA Lorenzo A. The evolution of physical and technical performance parameters in the Chinese soccer super league. Biol Sport. (2020) 37:139–45. 10.5114/biolsport.2020.93039 32508381 PMC7249799

[13] Lago-Penas C Lorenzo-Martinez M Lopez-Del Campo R Resta R Rey E. Evolution of physical and technical parameters in the spanish LaLiga 2012-2019. Sci Med Footb. (2022) 7:1–6. 10.1080/24733938.2022.2049980 35243954

[14] Nevill AM Okojie DI Smith J O'donoghue PG Webb T. Are professional footballers becoming lighter and more ectomorphic? Implications for talent identification and development. Int J Sports Sci Coach. (2019) 14:329–35. 10.1177/1747954119837710

[15] Haugen TA Tonnessen E Seiler S. Anaerobic performance testing of professional soccer players 1995-2010. Int J Sports Physiol Perform. (2013) 8:148–56. 10.1123/ijspp.8.2.148 22868347

[16] Elferink-Gemser MT Huijgen BC Coelho-E-Silva M Lemmink KA Visscher C. The changing characteristics of talented soccer players – a decade of work in Groningen. J Sports Sci. (2012) 30:1581–91. 10.1080/02640414.2012.725854 23020141

[17] Tonnessen E Hem E Leirstein S Haugen T Seiler S. Maximal aerobic power characteristics of male professional soccer players, 1989-2012. Int J Sports Physiol Perform. (2013) 8:323–9. 10.1123/ijspp.8.3.323 23118070

[18] Carling C Le Gall F Malina RM. Body size, skeletal maturity, and functional characteristics of elite academy soccer players on entry between 1992 and 2003. J Sports Sci. (2012) 30:1683–93. 10.1080/02640414.2011.637950 22292471

[19] Gonaus C Birklbauer J Lindinger SJ Stöggl TL Müller E. Changes over a decade in anthropometry and fitness of elite Austrian youth soccer players. Front Physiol. (2019) 10:333. 10.3389/fphys.2019.00333 30984022 PMC6447713

[20] Malina RM Eisenmann JC Cumming SP Ribeiro B Aroso J. Maturity-associated variation in the growth and functional capacities of youth football (soccer) players 13-15 years. Eur J Appl Physiol. (2004) 91:555–62. 10.1007/s00421-003-0995-z 14648128

[21] Gil SM Badiola A Bidaurrazaga-Letona I Zabala-Lili J Gravina L Santos-Concejero J Relationship between the relative age effect and anthropometry, maturity and performance in young soccer players. J Sports Sci. (2014) 32:479–86. 10.1080/02640414.2013.832355 24050650

[22] Bidaurrazaga-Letona I Lekue JA Amado M Gil SM. Progression in youth soccer: selection and identification in youth soccer players aged 13-15 years. J Strength Cond Res. (2019) 33:2548–58. 10.1519/JSC.0000000000001924 28394831

[23] Patel R Nevill A Smith T Cloak R Wyon M. The influence of birth quartile, maturation, anthropometry and physical performances on player retention: observations from an elite football academy. Int J Sports Sci Coach. (2020) 15:121–34. 10.1177/1747954120906507

[24] Parr J Winwood K Hodson-Tole E Deconinck FJA Hill JP Teunissen JW The main and interactive effects of biological maturity and relative age on physical performance in elite youth soccer players. J Sports Med. (2020):1–11. 10.1155/2020/1957636

[25] Gonaus C Müller E. “Test-retest reliability of a field-based fitness test battery used in Austrian youth soccer”. In: Balagué N Torrents C Vilanova A Cadefau J Tarragó R Tsolakidis E, editors. Book of abstracts of the 18th annual congress of the European college of sport science in Barcelona. Cologne: European College of Sport Science (2013). p. 146.

[26] Paul DJ Nassis GP. Physical fitness testing in youth soccer: issues and considerations regarding reliability, validity and sensitivity. Pediatr Exerc Sci. (2015) 27:301–13. 10.1123/pes.2014-0085 26331619

[27] Stratton G Reilly T Williams AM Richardson D. Youth soccer: From science to performance. London: Routledge (2004).

[28] Unnithan V White J Georgiou A Iga J Drust B. Talent identification in youth soccer. J Sports Sci. (2012) 30:1719–26. 10.1080/02640414.2012.731515 23046427

[29] Gonaus C Müller E. Using physiological data to predict future career progression in 14- to 17-year-old Austrian soccer academy players. J Sports Sci. (2012) 30:1673–82. 10.1080/02640414.2012.713980 22917159

[30] Drust B Waterhouse J Atkinson G Edwards B Reilly T. Circadian rhythms in sports performance—an update. Chronobiol Int. (2005) 22:21–44. 10.1081/CBI-200041039 15865319

[31] Svensson M Drust B. Testing soccer players. J Sports Sci. (2005) 23:601–18. 10.1080/02640410400021294 16195009

[32] Harman E. “Principles of test selection and administration,”. In: Baechle TR Earle RW, editors. Essentials of strength training and conditioning. 3rd ed Champaign, IL: Human kinetics (2008). p. 237–47.

[33] Hopkins WG Marshall SW Batterham AM Hanin J. Progressive statistics for studies in sports medicine and exercise science. Med Sci Sports Exerc. (2009) 41:3–13. 10.1249/MSS.0b013e31818cb278 19092709

[34] Thoemmes F. Propensity score matching in SPSS. (2012). Available: https://arxiv.org/abs/1201.6385 (Accessed 30.07.2019).

[35] Stuart EA. Matching methods for causal inference: a review and a look forward. Stat Sci. (2010) 25:1–21. 10.1214/09-STS313 20871802 PMC2943670

[36] Lunt M. Selecting an appropriate caliper can be essential for achieving good balance with propensity score matching. Am J Epidemiol. (2014) 179:226–35. 10.1093/aje/kwt212 24114655 PMC3873103

[37] Rosenbaum PR Rubin DB. Constructing a control-group using multivariate matched sampling methods that incorporate the propensity score. Am Stat. (1985) 39:33–8. 10.2307/2683903

[38] Caliendo M Kopeinig S. Some practical guidance for the implementation of propensity score matching. J Econ Surv. (2008) 22:31–72. 10.1111/j.1467-6419.2007.00527.x

[39] West SG Cham H Thoemmes F Renneberg B Schulze J Weiler M. Propensity scores as a basis for equating groups: basic principles and application in clinical treatment outcome research. J Consult Clin Psychol. (2014) 82:906–19. 10.1037/a0036387 24708350

[40] Schafer JL Kang J. Average causal effects from nonrandomized studies: a practical guide and simulated example. Psychol Methods. (2008) 13:279–313. 10.1037/a0014268 19071996

[41] Austin PC. A comparison of 12 algorithms for matching on the propensity score. Stat Med. (2014) 33:1057–69. 10.1002/sim.6004 24123228 PMC4285163

[42] Enders CK. Applied missing data analysis. New York: Guilford Press (2010).

[43] Lakens D. Calculating and reporting effect sizes to facilitate cumulative science: a practical primer for *t*-tests and ANOVAs. Front Psychol. (2013) 4:863. 10.3389/fpsyg.2013.00863 24324449 PMC3840331

[44] Cohen J. A power primer. Psychol Bull. (1992) 112:155–9. 10.1037/0033-2909.112.1.155 19565683

[45] Nassis GP Massey A Jacobsen P Brito J Randers MB Castagna C Elite football of 2030 will not be the same as that of 2020: preparing players, coaches, and support staff for the evolution. Scand J Med Sci Sports. (2020) 30:962–4. 10.1111/sms.13681 32424904

[46] Reilly T. Training specificity for soccer. Int J Appl Sports Sci. (2005) 17:17–25.

[47] Bujalance-Moreno P Latorre-Roman PA Garcia-Pinillos F. A systematic review on small-sided games in football players: acute and chronic adaptations. J Sports Sci. (2019) 37:921–49. 10.1080/02640414.2018.1535821 30373471

[48] Sheppard JM Young WB. Agility literature review: classifications, training and testing. J Sports Sci. (2006) 24:919–32. 10.1080/02640410500457109 16882626

[49] Chaouachi A Chtara M Hammami R Chtara H Turki O Castagna C. Multidirectional sprints and small-sided games training effect on agility and change of direction abilities in youth soccer. J Strength Cond Res. (2014) 28:3121–7. 10.1519/JSC.0000000000000505 25148467

[50] Querido SM Clemente FM. Analyzing the effects of combined small-sided games and strength and power training on the fitness status of under-19 elite football players. J Sports Med Phys Fitness. (2020) 60:1–10. 10.23736/S0022-4707.19.09818-9 32008309

[51] Douchet T Paizis C Carling C Cometti C Babault N. Typical weekly physical periodization in French academy soccer teams: a survey. Biol Sport. (2023) 40:731–40. 10.5114/biolsport.2023.119988 37398965 PMC10286623

[52] Dolci F Hart NH Kilding AE Chivers P Piggott B Spiteri T. Physical and energetic demand of soccer: a brief review. Strength Cond J. (2020) 42:70–7. 10.1519/SSC.0000000000000533

[53] Murr D Raabe J Höner O. The prognostic value of physiological and physical characteristics in youth soccer: a systematic review. Eur J Sport Sci. (2018) 18:62–74. 10.1080/17461391.2017.1386719 29161984

[54] Vestberg T Jafari R Almeida R Maurex L Ingvar M Petrovic P. Level of play and coach-rated game intelligence are related to performance on design fluency in elite soccer players. Sci Rep. (2020) 10:9852. 10.1038/s41598-020-66180-w 32587269 PMC7316809

[55] Huijgen BC Leemhuis S Kok NM Verburgh L Oosterlaan J Elferink-Gemser MT Cognitive functions in elite and sub-elite youth soccer players aged 13 to 17 years. PLoS One. (2015) 10:e0144580. 10.1371/journal.pone.014458 26657073 PMC4691195

[56] Goncalves E Noce F Barbosa MaM Figueiredo AJ Teoldo I. Maturation, signal detection, and tactical behavior of young soccer players in the game context. Sci Med Footb. (2021) 5:272–9. 10.1080/24733938.2020.1851043 35077304

[57] Henderson G Barnes CA Portas MD. Factors associated with increased propensity for hamstring injury in English premier league soccer players. J Sci Med Sport. (2010) 13:397–402. 10.1016/j.jsams.2009.08.003 19800844

[58] Garcia-Pinillos F Ruiz-Ariza A Moreno Del Castillo R Latorre-Roman PA. Impact of limited hamstring flexibility on vertical jump, kicking speed, sprint, and agility in young football players. J Sports Sci. (2015) 33:1293–7. 10.1080/02640414.2015.1022577 25761523

[59] Perez-Gomez J Adsuar JC Alcaraz PE Carlos-Vivas J. Physical exercises for preventing injuries among adult male football players: a systematic review. J Sport Health Sci. (2022) 11:115–22. 10.1016/j.jshs.2020.11.003 33188962 PMC8847925

[60] Lovell R Towlson C Parkin G Portas M Vaeyens R Cobley S. Soccer player characteristics in English lower-league development programmes: the relationships between relative age, maturation, anthropometry and physical fitness. PLoS One. (2015) 10:e0137238. 10.1371/journal.pone.0137238 26331852 PMC4558096

[61] Johnson A Farooq A Whiteley R. Skeletal maturation status is more strongly associated with academy selection than birth quarter. Sci Med Footb. (2017) 1:157–63. 10.1080/24733938.2017.1283434

[62] Vandendriessche JB Vaeyens R Vandorpe B Lenoir M Lefevre J Philippaerts RM. Biological maturation, morphology, fitness, and motor coordination as part of a selection strategy in the search for international youth soccer players (age 15-16 years). J Sports Sci. (2012) 30:1695–703. 10.1080/02640414.2011.652654 22296038

[63] Towlson C Macmaster C Parr J Cumming S. One of these things is not like the other: time to differentiate between relative age and biological maturity selection biases in soccer? Sci Med Footb. (2022) 6:273–6. 10.1080/24733938.2021.1946133 35866421

[64] Sturmer T Joshi M Glynn RJ Avorn J Rothman KJ Schneeweiss S. A review of the application of propensity score methods yielded increasing use, advantages in specific settings, but not substantially different estimates compared with conventional multivariable methods. J Clin Epidemiol. (2006) 59:437–47. 10.1016/j.jclinepi.2005.07.004 16632131 PMC1448214

[65] Thoemmes FJ Kim ES. A systematic review of propensity score methods in the social sciences. Multivariate Behav Res. (2011) 46:90–118. 10.1080/00273171.2011.540475 26771582

[66] Austin PC. An Introduction to propensity score methods for reducing the effects of confounding in observational studies. Multivariate Behav Res. (2011) 46:399–424. 10.1080/00273171.2011.568786 21818162 PMC3144483

[67] Beal SJ Kupzyk KA. An Introduction to propensity scores: what, when, and how. J Early Adolesc. (2014) 34:66–92. 10.1177/0272431613503215

[68] D'agostino RB. Propensity scores in cardiovascular research. Circulation. (2007) 115:2340–3. 10.1161/CIRCULATIONAHA.105.594952 17470708

[69] Turner A Walker S Stembridge M Coneyworth P Reed G Birdsey L A testing battery for the assessment of fitness in soccer players. Strength Cond J. (2011) 33:29–39. 10.1519/SSC.0b013e31822fc80a

